# Learning the shape of protein microenvironments with a holographic convolutional neural network

**DOI:** 10.1073/pnas.2300838121

**Published:** 2024-02-01

**Authors:** Michael N. Pun, Andrew Ivanov, Quinn Bellamy, Zachary Montague, Colin LaMont, Philip Bradley, Jakub Otwinowski, Armita Nourmohammad

**Affiliations:** ^a^Department of Physics, University of Washington, Seattle, WA 98195; ^b^The Department for Statistical Physics of Evolving Systems, Max Planck Institute for Dynamics and Self-Organization, Göttingen 37077, Germany; ^c^Fred Hutchinson Cancer Center, Seattle, WA 98102; ^d^Department of Biochemistry, University of Washington, Seattle, WA 98195; ^e^Institute for Protein Design, University of Washington, Seattle, WA 98195; ^f^Dyno Therapeutics, Watertown, MA 02472; ^g^Department of Applied Mathematics, University of Washington, Seattle, WA 98105; ^h^Paul G. Allen School of Computer Science and Engineering, University of Washington, Seattle, WA 98195

**Keywords:** protein science, protein structure–function map, machine learning, geometric deep learning, rotationally equivariant convolutional neural network

## Abstract

Proteins play a central role in biology from immune recognition to brain activity. While major advances in machine learning have improved our ability to predict protein structure from sequence, determining protein function from its sequence or structure remains a major challenge. Here, we introduce holographic convolutional neural network (H-CNN) for proteins, which is a physically motivated machine learning approach to model amino acid preferences in protein structures. H-CNN reflects physical interactions in a protein structure and recapitulates the functional information stored in evolutionary data. H-CNN accurately predicts the impact of mutations on protein stability and binding of protein complexes. Our interpretable computational model for protein structure–function maps could guide design of novel proteins with desired function.

## Significance

Proteins are the machinery of life facilitating the key processes that drive living organisms. Recent advances have increased the number of experimentally resolved or computationally predicted tertiary structures of proteins. However, we still lack an understanding of how tertiary structure determines the function of a protein. M. Pun et al. introduce a physically motivated machine learning approach to learn interpretable models for protein structures, reflecting the underlying biophysics. This model accurately predicts the impact of mutations on protein stability and binding of protein complexes. The flexibility and efficiency of this approach also show promise for building generative models to design novel protein structures with desired stability and binding reactivity.

Proteins are the machinery of life. They facilitate the key processes that drive living organisms and generally rely on only twenty amino acids to do so. Therefore, most chemical reactions in biological systems involve interactions between a protein’s residues and its atomic environment, including other proteins, small molecules, singular ions, or other biomolecules. Understanding the interactions between a given amino acid and its atomic environment is the key to understanding a protein’s physicochemical properties, including stability and binding interaction with other molecules.

With the growing amount of data and computational advances, machine learning has come to the forefront of protein science, especially in predicting structure from sequence (1–5). However, the problem of how a protein’s physical and chemical properties are determined from its sequence or structure still remains a major challenge.

Techniques from natural language processing are used to determine functional motifs in protein sequences by allowing residues far away in sequence to form information units about function (6–12). However, since a protein’s stability and function are closely related to protein structure, models trained to predict these properties from protein sequences at least implicitly account for the complex sequence–structure map.

Despite AlphaFold’s remarkable success at predicting protein folding, it still struggles to determine the effect of mutations on the stability and function of a protein (13). Nonetheless, it is suggested that AlphaFold has learned an effective physical energy potential to fold proteins, and therefore, it could be used to characterize the effect of mutations or general protein function (14). Given the availability of high-resolution tertiary structures, obtained either experimentally or computationally, the information on the 3D atomic composition of a protein can be used to learn various physicochemical properties of proteins.

Structure-based models, and in particular those that represent the atomic components of proteins, have been shown to be successful at protein tasks such as rotamer packing (15), sequence design (16), energy prediction (17), and stability prediction (18). Despite the use of structure in these methods, not all prioritize the geometric symmetries that are natural to the atomic composition in the protein structure.

Accounting for the geometry of a protein 3D structure can enable machine learning models to reason about physical interactions within a protein, resulting in more data-efficient models with cross-task generalization ability. For example, geometry-aware structure-based models that attempt to solve the inverse protein folding problem, i.e., designing a sequence that folds into a desired structure, can be used to reliably infer the functional effect of mutations in a protein sequence (19), or even engineer diverse sequences that have a desired function (20).

Recent work in the field of molecular dynamics (MD) has shown the power of geometry-aware machine learning at inferring precise interatomic force fields (21–28). Compared to the geometry-aware protein structure models (3, 4, 19, 20), the MD models use more complex geometric features, resulting in more expressive, yet physically interpretable models of molecular interactions.

**Fig. 1.**
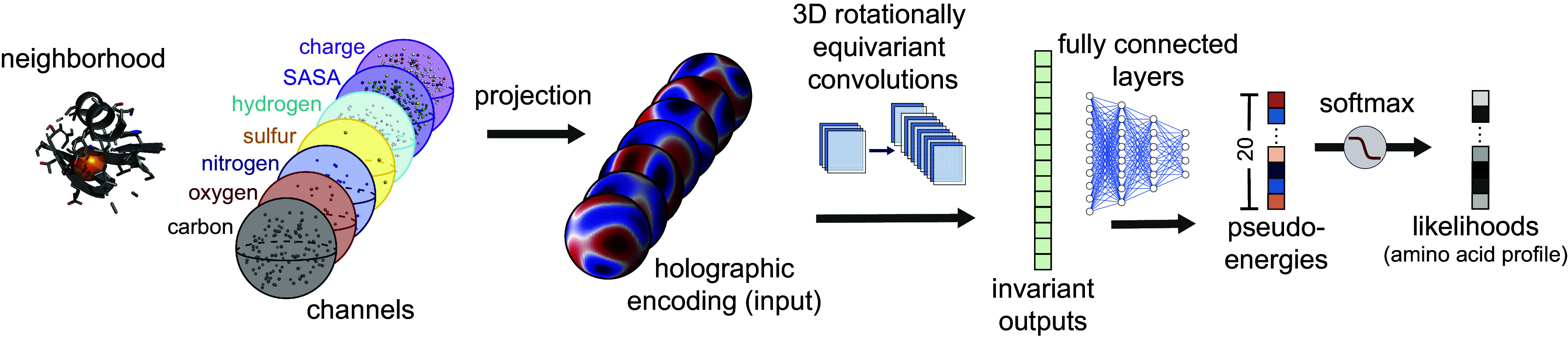
Schematic of holographic convolutional neural network (H-CNN) for protein microenvironments. A neighborhood within a radius of 10 Å around a focal amino acid (masked in orange) in a protein structure is separated into its constituent atomic and chemical channels. The information in these channels is encoded in a rotationally equivariant form, using 3D Zernike polynomials, which defines holograms in spherical Fourier space. These holographic encodings are processed by a rotationally equivariant convolutional neural network Clebsch–Gordan net (29). The invariant features of the network layers are then collected and processed through fully connected feed-forward layers to determine the preferences, i.e., statistical weights (pseudoenergies) and probabilities, for different amino acids residing at the center of the input neighborhood. The set of predicted probability vectors across all 20 amino acids defines an amino acid profile. The network is trained by learning the categorical classification task with a softmax cross-entropy loss on a one-hot label of the neighborhood determined by the true central residue in the protein structure. A more detailed network architecture is presented in *SI Appendix*, Fig. S2.

Here, we introduce geometry-aware holographic convolutional neural network (H-CNN) to learn physically grounded models for protein structures that can be used to predict the impact of different amino acids on physicochemical properties of a protein. H-CNN learns local representations of protein structures by encoding the atoms within protein microenvironments in a spherical Fourier space as holograms, and processes these holographic projections via a 3D rotationally equivariant convolutional neural network (Fig. 1) (29–31). The resulting model respects rotational symmetry of protein structures and characterizes effective inter-amino acid potentials in protein microenvironments.

We train H-CNN on protein structures available in the Protein Data Bank (PDB) (32) and perform the supervised task of predicting the identity of an amino acid from its surrounding atomic neighborhood with a high accuracy and computational efficiency. The amino acids that H-CNN infers to be interchangeable have similar physicochemical properties, and the pattern is consistent with substitution patterns in evolutionary data. The H-CNN model encodes a more complete set of geometric features of protein structures compared to the other geometry-aware models of proteins (12, 19, 20). Therefore, it can predict the impact of mutations on protein stability and binding, only based on its local atomic composition within a protein structure. Our results showcase that principled geometry-aware machine learning can lead to powerful and robust models that provide insight into the biophysics of protein stability and function, with a potential for protein design.

## Results

### Model.

We define the microenvironment surrounding an amino acid as the identity and the 3D coordinates of atoms within a radius of 10 Å of the focal amino acid’s α-carbon; this neighborhood excludes atoms from the focal amino acid.

A common approach to encode such atomic neighborhoods for computational analysis is to voxelize the coordinates, which is a form of binning in 3D (33, 34). However, this approach distorts the information, since the voxel boundaries are arbitrary—too large voxels average over many atoms, and too small voxels lead to very sparse data.

The other obstacle to modeling such data is more fundamental and related to the rotational symmetries in encoding a protein structure neighborhood. A given neighborhood can occur in different orientations within or across proteins, and a machine learning algorithm should account for such rotational symmetry. One approach known as data augmentation, mainly used in image processing, trains an algorithm on many examples of an image in different orientations and locations. Data augmentation is computationally costly in 3D, and it is likely to result in a model of amino acid interactions that depends on the neighborhood’s orientation, which is a nonphysical outcome. Another approach is to orient the amino acid neighborhoods based on a prior choice (e.g., along the backbone of the protein) (33, 34). However, this choice is somewhat arbitrary, and the specified orientation of the protein backbone could inform the model about the identity of the focal amino acid.

To overcome these obstacles, we introduce the holographic convolutional neural networks (H-CNN) for protein microenvironments. H-CNN takes as input the coordinates, the atomic information (i.e., element type: carbon, nitrogen, oxygen, sulfur, hydrogen), and the physicochemical properties, i.e., solvent accessible surface area (SASA) and charge of all atoms within a 10 Å distance of the central residue’s α−carbon. This information is stored as point clouds in different input channels of H-CNN.

We use 3D Zernike polynomials as spherical basis functions to encode the information on different atom types and physicochemical properties associated with a given point cloud (Fig. 1, *Materials and Methods*, and *SI Appendix*). 3D Zernike polynomials can be used to expand any function in three dimensions along angular and radial bases and can uniquely represent the properties of the encoded object in a spherical Fourier space, given enough terms in the Fourier series. Conveniently, the angular components of the Zernike polynomials are spherical harmonics, which form an equivariant basis under rotation in 3D. Rotational equivariance is the property that if the input (i.e., atomic coordinates of an amino acid’s neighborhood) is rotated; then, the output is transformed according to a linear operation determined by the rotation (*SI Appendix*, Fig. S1). As a result, these Zernike projections enable us to encode the atomic point clouds from a protein structure without having to align the neighborhoods. Zernike projections in spherical Fourier space can be understood as a superposition of spherical holograms of an input point cloud, and thus, we term this operation as holographic encoding of protein microenvironments; see Fig. 1 and *SI Appendix*, Fig. S2 and *SI Appendix* for details.

The holograms encoding protein neighborhoods are input to a type of convolutional neural network (CNN). This network is trained on the supervised task of predicting the identity of a focal amino acid from the surrounding atoms in the protein’s tertiary structure. Conventional CNNs average over spatial translations and can learn features in the data that may be in different locations (i.e., they respect translational symmetry). For the analysis of protein neighborhoods, we need to infer models that are insensitive to the orientation of the data (i.e., they respect 3D rotational symmetry of the point clouds in a protein neighborhood).

Recent work in machine learning has expanded CNNs to respect physical symmetries beyond translations (29–31). For 3D rotations, generalized convolutions use spherical harmonics, which arise from the irreducible representations of the 3D rotation group SO(3) (35). For our analysis, we use Clebsch–Gordan neural networks (29), in which the linear and the nonlinear operations of the network layers have the property of rotational equivariance; see *Materials and Methods* and *SI Appendix* for details, *SI Appendix*, Fig. S2 for detailed information on network architecture, *SI Appendix*, Fig. S3 and Table S1 for details on hyperparameter tuning and training of the network.

The output of the trained model is a 20-dimensional vector of probabilities associated with the preference for having each of the 20 amino acids at the center of a given structural neighborhood. The logarithm of these probabilities can be interpreted as energies, since they parametrize the model’s distribution of amino acids at a given site. Specifically, we term the logits given by H-CNN as pseudoenergies, which are equal to the logarithm of probabilities up to a constant (Fig. 1). We will show in subsequent sections that these pseudoenergies are closely related to the experimentally determined free energy contributions of amino acids in proteins. Nonetheless, we use the “pseudo” prefix because these values do not have units of energies, and therefore, should be distinguished.

Taken together, the H-CNN shown in Fig. 1 and *SI Appendix*, Fig. S2 takes as input holograms that encode the spatial composition of different atoms and physical properties such as charge and SASA. The input is processed by a 3D rotationally equivariant CNN to learn statistical representations for protein neighborhoods. We train this H-CNN as a classifier on protein neighborhoods, collected from tertiary structures from the PDB, and use the trained network to quantify the preferences for different amino acids in a given structural neighborhood. For robustness, models were trained on both crystal structures and noised coordinates; see *Materials and Methods* and *SI Appendix* for details on data preprocessing.

### H-CNN Reveals Physicochemical Properties of Amino Acids, Consistent with Evolutionary Variation.

**Fig. 2.**
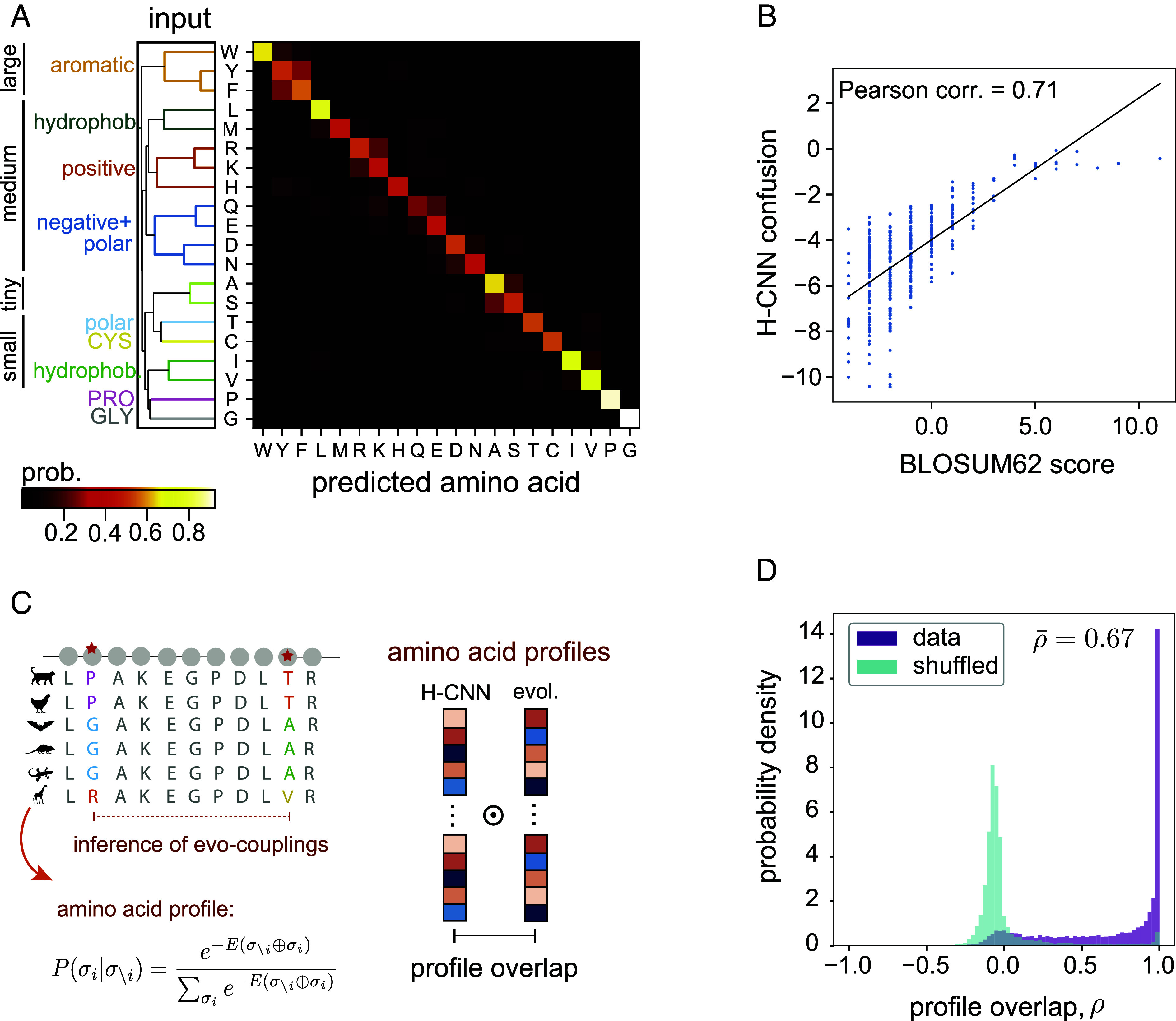
H-CNN predicts amino acid preferences in protein microenvironments. (*A*) The confusion matrix for amino acid predictions with H-CNN shows the mean H-CNN predicted probabilities of each of the twenty amino acids (output) conditioned on a specific central amino acid (input). Overall prediction accuracy is 68%. The hierarchical clustering for these predictions reflects known similarities in size and physicochemical properties of amino acids. (*B*) Amino acid confusion in (*A*) correlates with the substitutability of amino acids in natural proteins as determined by the BLOSUM62 matrix; 71% Pearson correlation. (*C*) Schematic shows how evolutionary covariation of amino acids in multiple sequence alignments of protein families can be used to fit Potts models EV-couplings (36) to characterize the probability of a given amino acid, given the rest of the sequence (*Left*); see *SI Appendix* for details. To compare evolutionary and H-CNN predictions for site-specific amino acid profiles, the profile overlap is computed as the centered cosine similarity between the predicted probability profiles (*Right*); see *SI Appendix*. (*D*) The profile overlaps are strongly peaked around one, implying perfect overlap in data (purple); the average profile overlap across 11,221 sites from a total of 67 protein families is $\overset{¯}{\rho }=0.67$. The H-CNN predictions are notably different for the shuffled data, for which the profile overlap peaks near zero (cyan), with an average of 0.002.

H-CNN predicts the identity of an amino acid from its surrounding microenvironment with 68% accuracy (Fig. 2). Notably, our results are robust to different splittings that restrict the degree of sequence or structural similarities between training and test sets (*Materials and Methods*, *SI Appendix*, and *SI Appendix*, Fig. S4).

The accuracy of H-CNN is comparable to state-of-the-art approaches with conventional CNNs that voxelize and orient the data along the backbone of a central amino acid, while using a smaller atomic microenvironment for performing this classification task (33, 34); see Table 1 for a detailed comparison of models and *SI Appendix* for information on alternative models. Notably, restricting the training of H-CNN to the subspace of models that are rotationally equivariant leads to a substantial speedup in the training of H-CNN compared to the conventional techniques (33, 34). Moreover, H-CNN is more accurate than other symmetry-aware approaches for molecular modeling (37, 38), while using an order of magnitude fewer parameters; see Table 1 and *SI Appendix* for a detailed comparison of models.

**Table 1.** Comparison of structure-based models for amino acid retrieval in protein neighborhoods

Method	Rotationally invariant	Dataset	Postprocessing	Scale	N	No. of parameters	Training time	Accuracy, %
H-CNN	Yes	ProteinNet	Charge hydrogen	d=20Å	$2.8\times 10^{6}$	$3.6\times 10^{6}$	4.54 h	68
		CATH 4.2	SASA		$3.3\times 10^{6}$			
3D DNN (Torng) (33)	No	SCOP & ASTRAL	None	ℓ=20Å	$7.2\times 10^{5}$	$10^{7}$	3 d	40
3D CNN (Shroff) (34)	No	SCOP & ASTRAL	PDB-REDO (39) charge hydrogen SASA	ℓ=20Å	$1.6\times 10^{6}$	$6.1\times 10^{7}$	–	70
Spherical CNN (37)	Approx.	PISCES	Charge hydrogen	d=24Å	–	$6\times 10^{7}$	–	56
Spherical CNN Rasp (18)	Approx.	PISCES	Hydrogen OpenMM PDBFixer	d=18Å	–	–	–	63
Steerable CNN (38)	Yes	SCOP & ASTRAL	PDB-REDO charge hydrogen SASA	d=24Å	$1.6\times 10^{6}$	$3.3\times 10^{7}$	–	58
Protein MPNN (20)	Yes	CATH 4.2	–	Entire protein backbone	–	–	–	52.4

H-CNN and existing methods trained to classify residues from the surrounding neighborhoods are listed along with the available information and summary statistics of the models. With the exception of ProteinMPNN, which is trained solely on backbone atoms, all other methods are all-atom-based. The scale for each model represents the size of the atomic neighborhood each model uses to the predict central amino acid class. For models that use cubic volumes, the side length ℓ is reported, while for models that use spherical volumes, the diameter d is reported. H-CNN demonstrates the power of respecting symmetry since it has fewer parameters and trains faster than 3D-CNNs despite being trained on at least the same amount of data.

H-CNN predicts the conformationally unique amino acids of Glycine and Proline with over 90% accuracy. Meanwhile, amino acids with typical side-chains cluster based on their sizes and the physicochemical properties of the side-chains including aromatic, hydrophobic, and charged groupings (Fig. 2*A*). The inferred amino acid preferences cluster well according to the input amino acid type (true label) in the low-dimensional UMAP representation (40), and amino acids with similar physicochemical properties cluster in nearby regions in the UMAP (*SI Appendix*, Fig. S5).

H-CNN predictions reflect amino acid preferences seen in evolutionary data, even though the network is not trained on multiple sequence alignments (MSAs) of protein homologs. Specifically, the interchangeability of amino acids that H-CNN predicts is 71% correlated with the substitution patterns in evolutionary data, represented by the BLOSUM62 matrix (Fig. 2*B*). In addition, the amino acid preferences predicted by H-CNN at each site are consistent with evolutionary preferences inferred from the covariation of residues in multiple sequence alignments of protein families (36, 41, 42); see Fig. 2 *C* and *D* and *SI Appendix* for details.

We tested the robustness of H-CNN by evaluating its retrieval accuracy on noised structures, by adding a Gaussian noise with varying amplitudes (i.e., SD) to the atomic coordinates of original neighborhoods; see *SI Appendix* for details on the noising procedure. H-CNN’s recovery of the true central amino acid deteriorates as noise is added to the structures in the test data (*SI Appendix*, Fig. S6*A*). The low-frequency cutoff in the Fourier projection of our holographic encoding blurs atomic coordinates, which could help to prevent overfitting on the exact shape of vacancies of the masked central residues in protein neighborhoods. Nonetheless, the model’s sensitivity to noise in the testing structures reflects that H-CNN may still use the exact shape to recover amino acid identity of the central residue.

To prevent the direct use of shape, we trained H-CNN on structural neighborhoods noised to various extents. Notably, when tested on noised data (with amplitude of 0.5 Å), networks trained on data with a noise amplitude of 0.3 to 0.5 Å show more than 10% improvement in retrieval accuracy, compared to those trained on unnoised data (*SI Appendix*, Fig. S6*B*). We later show that these models offer better predictions for the stability effect of mutations. Indeed, the use of noise to build more robust models has shown success both in protein-specific neural networks (20) as well as machine learning at large (43).

Last, we assessed the importance of the physicochemical properties (i.e., SASA and charge) for model performance. Specifically, we performed ablation studies where we trained H-CNN without the SASA and charge inputs, which resulted in roughly a 10% drop in classification accuracy; see *SI Appendix*, Fig. S7 and the discussion on ablation studies in *SI Appendix*. Notably, information from SASA mostly impacts the network’s ability to predict hydrophobic amino acids, with some hydrophilic amino acids (R, K, E) also impacted. When charge is removed, the network demonstrates worse predictions on charged and polar amino acids, most notably R, C, N, and E. These ablation studies further reveal that the H-CNN’s processing of information corresponds to physical intuition.

### H-CNN Learns an Effective Physical Potential for Protein Microenvironments.

**Fig. 3.**
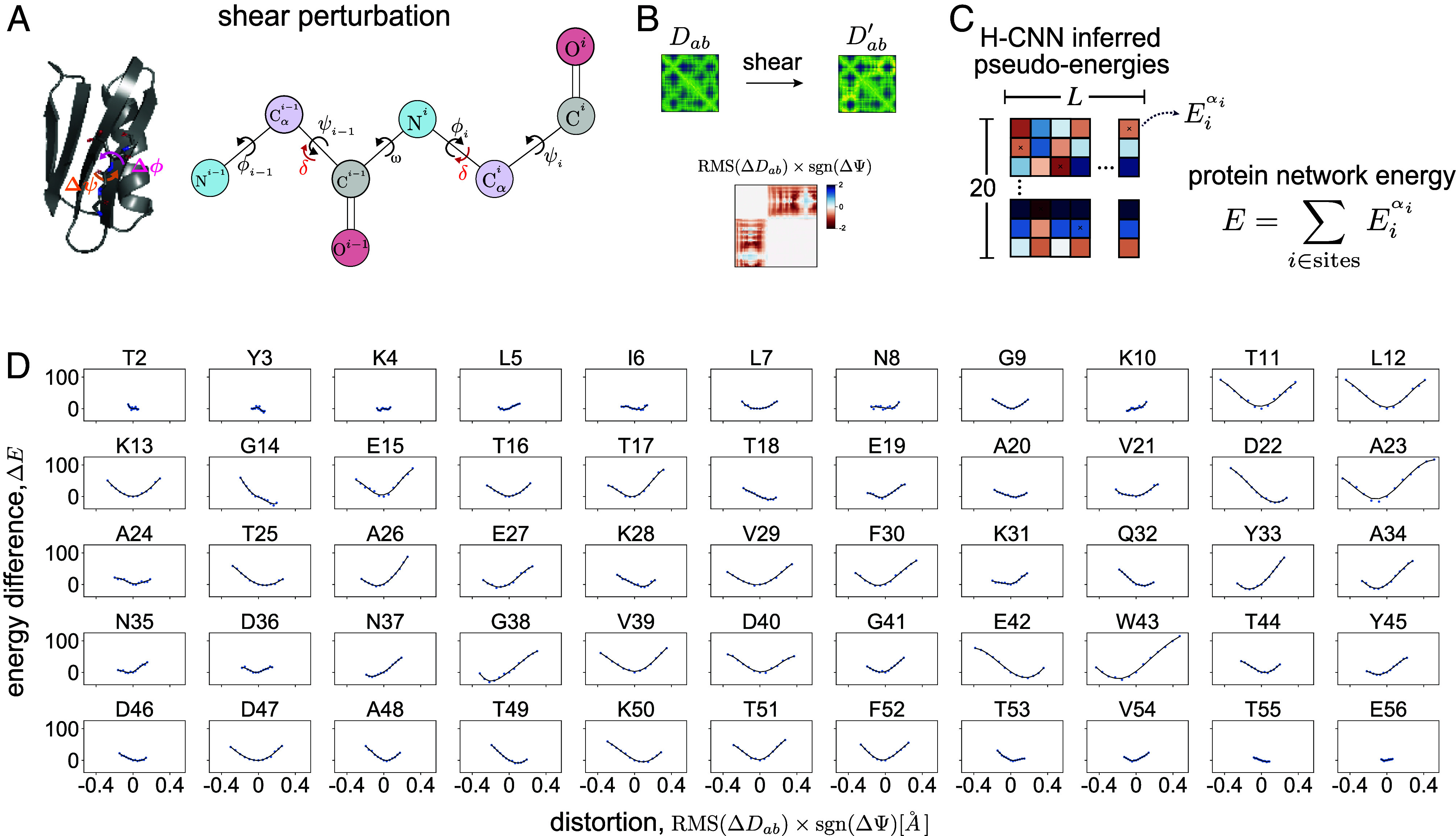
Response of H-CNN predictions to physical distortions in protein structures. (*A*) The schematic shows shear perturbation in a protein backbone by an angle δ at site i as a rotation of side-chains around the backbone by the angles $\left[\phi_{i},\psi_{i-1}\right]\to \left[\phi_{i}+\delta ,\psi_{i-1}-\delta \right]$ (32). (*B*) Shearing changes the pairwise distance matrix $D_{\mathrm{ab}}$ between all atoms in a protein structure. The total physical distortion is computed as the Rms of changes in the pairwise distances that are less than 10 Å (i.e., residues within the same neighborhood), multiplied by the sign of the change in the angle ψ. (*C*) For a given perturbation, the network energy E is determined by the sum of pseudoenergies of the wild-type amino acid at all sites in the protein, and the change in this quantity by shearing ΔE measures the tolerance of a structure to a given perturbation. (*D*) Panels show the change in the network energy in response to the structural distortion by shear perturbation at all sites in protein G, with the amino acid type and the site number indicated above each panel.

Since H-CNN is trained to predict the most natural amino acid given its neighborhood, it should also be able to recognize an unnatural protein configuration. To test this hypothesis, we characterize the response of the H-CNN predictions to physical distortions in native atomic microenvironments. We introduce distortions through local in silico shear perturbation of the protein backbone at a given site i by an angle δ, resulting in a transformation of the backbone angles by $\phi_{i}\to \phi_{i}+\delta ,\;\psi_{i-1}\to \psi_{i-1}-\delta$ (Fig. 3*A* and *SI Appendix*). In these shear perturbations, we twist the backbone angles by $\delta \;\leq \;20^{{}^{\circ}}$, corresponding to a less than 0.4 Å Rms deviation of the pairwise distances. This perturbation can substantially change the local protein structure near the residue of interest, while minimally affecting the far-away residues (32).

We measure the distortion of the protein structure due to shear by calculating the change in the Rms deviation in the pairwise distances of all atoms of the perturbed protein structure relative to that of the wild-type ($\text{RMS}\Delta D_{\mathrm{ab}},\;\;\text{for all pairs of atoms}\;\left(a,b\right)$); Fig. 3*B*.

We measure the response of the protein to shear perturbation by analyzing the change in H-CNN predicted pseudoenergies $E_{i}^{\alpha }$ (i.e., the logits produced by H-CNN); see Fig. 1. Specifically, for a distorted structure with a specific choice of δ, we re-evaluate the pseudoenergy of each amino acid in the protein, and define the total H-CNN predicted energy by summing over the pseudoenergies of all the amino acids in a protein (Fig. 3*C*). The change in the predicted energy of a protein due to distortion (relative to the wild-type) ΔE is a measure of H-CNN’s response to a given perturbation. A positive ΔE indicates an unfavorable change in the protein structure.

We carried out this procedure on protein G (PDB ID: 1PGA), which is relatively small with only 56 residues, allowing for easy perturbation of all sites. The change in the predicted energy ΔE as a function of distortion in the structure $\;\text{RMS}\Delta D_{\mathrm{ab}}$ due to shearing at different sites reveals two trends (Fig. 3*D*). First, the protein network energy appears to respond locally quadratically to perturbations. Second, perturbations generally result in higher protein network energy, corresponding to a less favorable protein microenvironment. Taken together, by training on a classification task and by constraining the network to respect the relevant rotational symmetry, H-CNN has learned an effective physical potential for protein microenvironments in which the native structure is generally more favorable and robust to local perturbations (i.e., it is at the energy minimum).

This observation of a minimum energy extends beyond the wild-type sequence when biophysically similar amino acids are substituted in the energy sum (*SI Appendix* and *SI Appendix*, Fig. S8*A*). Notably, this pattern appears not to be just an artifact of the structure since the minimum disappears when random amino acids are used to calculate the network energy (*SI Appendix*, Fig. S8*B*).

### H-CNN Predicts Effect of Mutations on Protein Stability.

Characterizing amino acid preferences in a protein neighborhood is closely related to the problem of finding the impact of mutations on protein stability. Here, we test the accuracy of H-CNN in predicting the stability effect of mutations in 40 different variants of the T4 lysozyme protein. Each of these variants is one amino acid away from the wild-type, with variations spanning 23 residues of the protein. Notably, the tertiary structure of the wild-type T4 lysozyme protein as well as the 40 mutants are available through different studies (33, 44–61); see *SI Appendix*, Table S2 for details on these mutants.

**Fig. 4.**
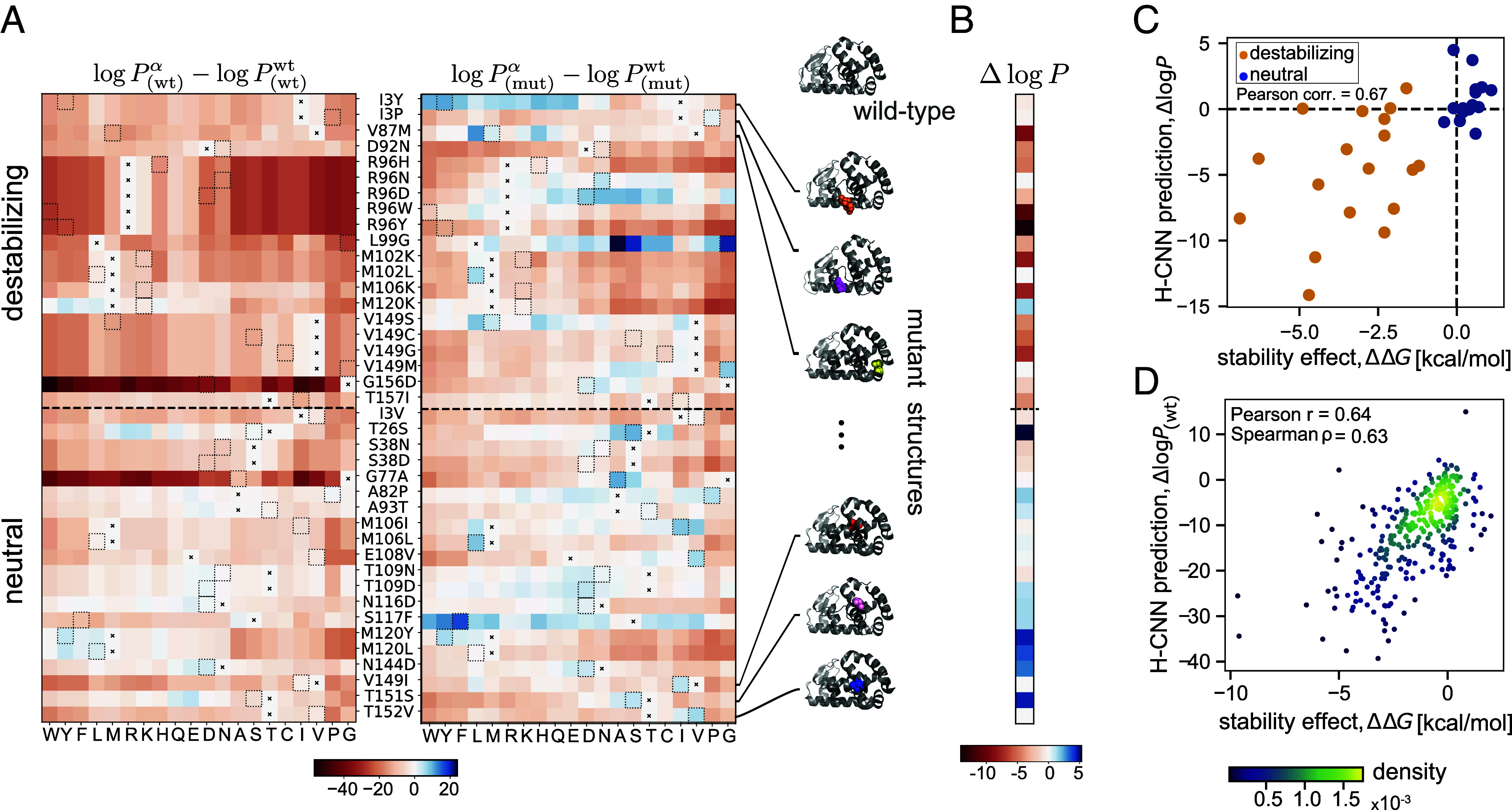
Predicting the stability effect of mutations in T4 lysozyme with H-CNN. (*A*) Heatmaps of H-CNN predicted log probability of different amino acids (columns) relative to that of the wild-type amino acid for 40 variants with single amino acid substitution from the wild-type (rows). For each variant (row), the position and the identity of the wild-type amino acid and the mutation are denoted between the two heatmaps as: wild-type, site number, mutation. The *Left* panel shows the predictions using the wild-type protein structure subscript (wt), while the *Right* panel shows the predictions using the structure of the specified mutant at each row subscript (mut). In each row, the wild-type amino acid is indicated by an ×, and a dotted box shows the amino acid of the mutant. (*B*) Shown are the H-CNN predicted log-probability ratios $\Delta \log P=\log P_{\left(\mathrm{mut}\right)}^{\mathrm{mut}}/P_{\left(\mathrm{wt}\right)}^{\mathrm{wt}}$ for all 40 mutations, measuring the difference between the predicted log-probability of a mutant amino acid on the associated structure $\log P_{\left(\mathrm{mut}\right)}^{\mathrm{mut}}$, and that of the wild-type amino acid on the wild-type structure $P_{\left(\mathrm{wt}\right)}^{\mathrm{wt}}$. This log-probability ratio should be closely related to the stability effect of mutations ΔΔG. Accordingly, the predicted ratios for destabilizing mutations are negative, while those for the neutral/beneficial mutations are positive. (*C*) The H-CNN predicted log-probability ratio ΔlogP shown against the experimentally evaluated ΔΔG for the stability effect of mutations in each protein structure; Pearson correlation of 67%. (*D*) The H-CNN predictions for the relative log-probabilities $\Delta \log P_{\text{wt}}$ using the wild-type structure only are shown against the experimentally measured ΔΔG values for 310 single point mutation variants of T4 lysozyme. Mean ΔΔG was used when multiple experiments reported values for the same variant. The colors show the density of points as calculated via Gaussian kernel density estimation. The predictions are accurate with correlations indicated in the panel.

H-CNN predicts that the wild-type amino acids are the most favorable in the wild-type structure, while the mutant amino acids are generally more favorable in the mutant structures, regardless of their stabilizing effects (Fig. 4*A*). These variant-specific preferences are not surprising since the folded protein structure can relax to accommodate for amino acid changes, resulting in a structural neighborhood that is more consistent with the statistics of the microenvironments around the mutated amino acid than that of the wild-type. However, the confidence that H-CNN has in associating an amino acid with a given structural neighborhood can change depending on the stability effect of the mutation. The log-ratio of the H-CNN inferred probability for the mutant amino acid in the mutant structure versus that of the wild type amino acid in the wild type structure, $\Delta \log P=\;\log P_{\text{mut}}/P_{\text{wt}}$, can provide an approximation to the ΔΔG associated with the stability of a mutation (*SI Appendix*).

The inferred H-CNN predicted log-probability ratio is generally negative for destabilizing mutations, and nonnegative for neutral/weakly beneficial mutations (Fig. 4*B*). Previously, a structure-based CNN model with voxelized protein structures has shown a similar qualitative result (33). Further quantitative analysis shows that the log-probability ratio is 67% correlated with the experimentally evaluated ΔΔG values for these variants (Fig. 4*C*). Moreover, the receiver-operating-characteristic (ROC) curves in *SI Appendix*, Fig. S9*A* show that the log-ratio of amino acid probabilities can reliably discriminate between destabilizing and neutral mutations, with an area under the curve (AUC) of 0.90.

The availability of tertiary structures for a large number of variants is a unique feature of this dataset, and in most cases, such structural resolution is not accessible. To overcome this limitation and predict the stability effect of mutations by relying on the wild-type structure alone, we used PyRosetta to relax the wild-type T4 lysozyme structure around a specified amino acid change (32) (*SI Appendix*). We find that the log-probability ratios $\Delta \log\tilde{P}$ estimated based on these in silico relaxed mutant structures are mostly negative (nonnegative) for destabilizing (neutral) mutations (*SI Appendix*, Fig. S10) and are correlated with the stability effect of mutations ΔΔG (*SI Appendix*, Fig. S10). However, structural relaxation can add noise to the data, causing the protein microenvironments to deviate from the natural structures that H-CNN is trained on. Thus, using the in silico relaxed structures slightly reduces the discrimination power of our model between deleterious and near-neutral mutations (AUC = 0.83); see *SI Appendix*, Fig. S9*A*.

In contrast, the preferences estimated based on the wild-type structure only can discriminate between destabilizing and neutral mutations very well, even though most mutations are inferred to be deleterious with respect to the wild-type (AUC = 0.93 in *SI Appendix*, Fig. S9). In other words, by using the wild-type structure only, our model can predict the relative stability effect of mutations correctly but not the sign of ΔΔG (*SI Appendix*, Figs. S9 and S10). Indeed, our inferred log-probability ratios based on the wild-type structure show a substantial correlation of 64% (Pearson correlation) with the stability effect of a much larger set of 310 single point mutants (62), for which protein structures are not available (Fig. 4*D*).

When no experimentally determined structure is available, computationally resolved protein structures from AlphaFold can also be used to predict the stability effect of mutations. The H-CNN predictions using the template-free AlphaFold2 predicted structure of T4 lysozyme wild-type sequence display substantial discrimination ability between destabilizing and near-neutral mutations (*SI Appendix*, Fig. S9) and are correlated with the mutants’ ΔΔG values (*SI Appendix*, Figs. S9 and S10).

Interestingly, the H-CNN predictions for stability effect of mutations improve both as model predictions were ensembled as well as with an increase in the training noise injected (Table 2, and *SI Appendix*, Fig. S11). By averaging over predictions from an ensemble of 10 best networks trained on data with 0.5Å noise amplitude, we achieve a 83% correlation with the experimentally evaluated ΔΔG values, using only the wild-type structure, and 81% correlation, using both the wild-type and the mutant structures of the 40 T4 lysozyme variants. These noisy and ensembled H-CNN predictions are the state of the art for zero-shot predictions of stability effects, when compared to the three other available structure-based models, MutCompute (34), Spherical CNN used in the RaSP software (18), and ProteinMPNN (20); see Table 2 and *SI Appendix* for more details on these alternative models. Spherical CNN shows a comparable performance to H-CNN, when using both the wild-type and the mutant structures of T4 Lysozyme.

**Table 2.** Comparison of structure-based models for zero-shot prediction of mutational effects

Method	T4 $\Delta \log P_{\mathrm{wt}}$	T4 ΔlogP	SARS-CoV-2 bind AUC
	Pearson r | Spearman ρ	Pearson r | Spearman ρ	
H-CNN (best, unnoised)	0.66, 0.71	0.67, 0.70	0.77
H-CNN-0.02 ensembled	0.74, 0.74	0.74, 0.77	0.78
H-CNN-0.1 ensembled	0.76, 0.75	0.74, 0.76	0.77
H-CNN-0.3 ensembled	0.76, 0.74	0.75, 0.79	0.78
H-CNN-0.5 ensembled	**0.83**, 0.78	**0.81**, **0.80**	0.76
MutCompute (34)	0.67, 0.60	0.59, 0.60	**0.82**
Spherical CNN RaSP (18)	0.76, **0.79**	**0.81**, **0.80**	0.78
ProteinMPNN (20)	0.68, 0.73	0.76, 0.76	0.81

**Fig. 5.**
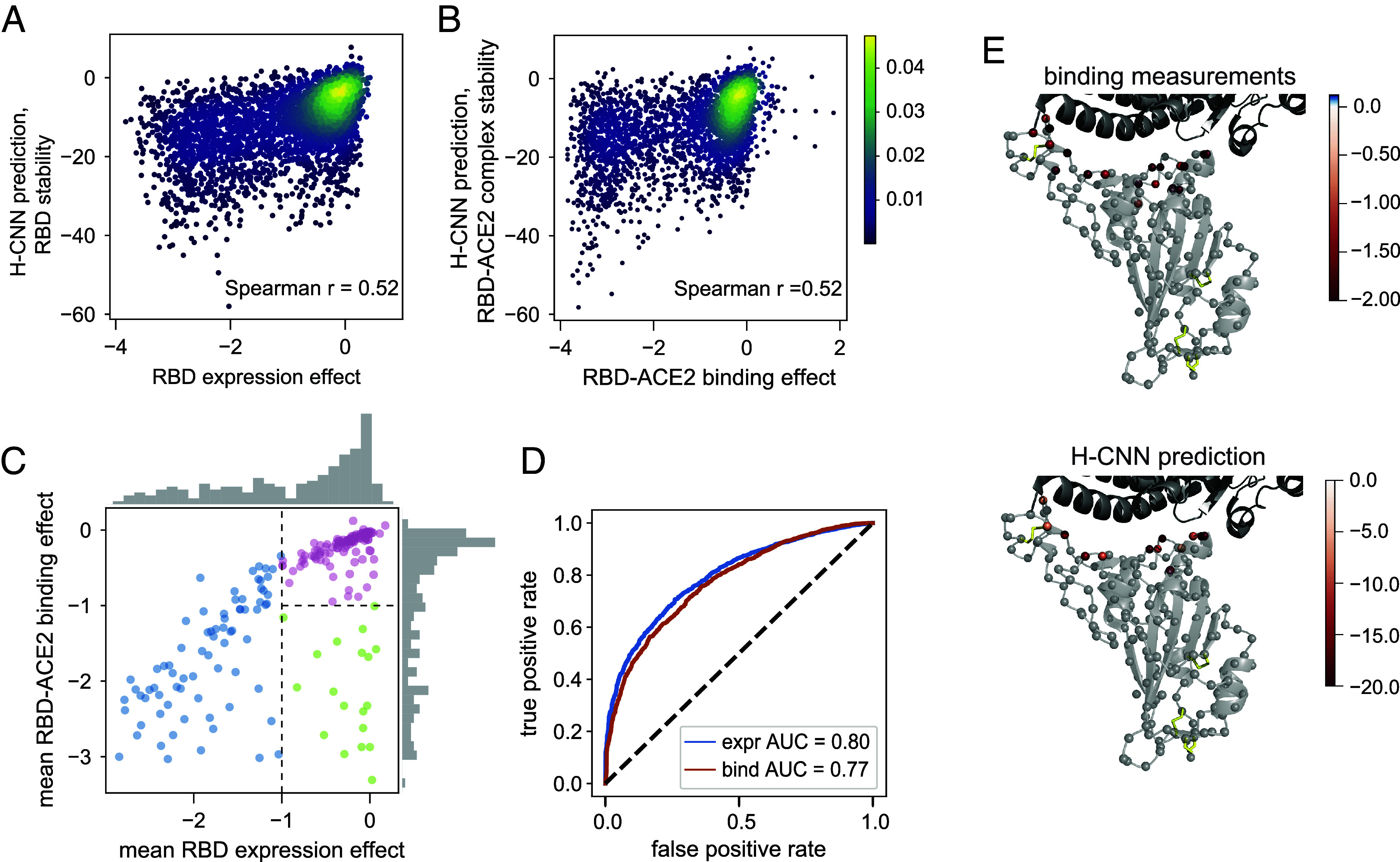
Predicting the stability and binding of the RBD protein of SARS-CoV-2 with H-CNN. (*A*) The density plot shows H-CNN predictions for the RBD stability, using the isolated protein structure of RBD, against the mutational effects on the RBD expression from the DMS experiments; Spearman correlation r=0.52. (*B*) The density plot shows H-CNN predictions for the RBD binding to the ACE2 receptor, using the cocrystallized RBD-ACE2 protein structure, against the DMS measurements for mutational effects on binding; shared color bar for (*A*) and (*B*). (*C*) The mean effect of mutations at each site on the RBD-ACE2 binding is shown against the mean effect on the RBD expression. The histograms show the corresponding distribution of effects across sites along each axis. The categories are shown: i) sites that are intolerant to mutations due to destabilizing effect, i.e., low expression (blue), ii) sites that are tolerant of mutations for expression but not binding (green), and iii) sites that are tolerant of mutations for both expression and binding (pink). (*D*) Blue: true positive vs. false positive rate (ROC curve) for classification of amino acid mutations into stable (expr >−1) vs. unstable (expr <−1), based on the H-CNN predictions using the isolated RBD structure; AUC = 0.8. Red: the ROC curve for mutation classification into bound (bind >−1) vs. unbound (bind <−1), based on the H-CNN predictions using the cocrystallized RBD-ACE2 structure; AUC = 0.74. (*E*) The effect of mutations on binding from the DMS experimental data for the green sites in (*C*, *Top*) and the corresponding H-CNN predictions from the RBD-ACE2 structure complex for sites identified by H-CNN in *SI Appendix*, Fig. S12 to be tolerant of mutations for stability but not binding (*Bottom*) are shown throughout the structure.

Performance of H-CNN, MutCompute (34), Spherical CNN used in the RaSP software (18), and ProteinMPNN (20) are compared on zero shot predictions of mutational effects on the stability of T4 Lysozyme and the binding of SARS-CoV-2 RBD to the human ACE2 receptor. Values are reported for the best H-CNN network trained on unnoised data, and H-CNN trained on noised data with varying amplitudes (indicated after the dash line). For noisy models, the reported values are estimated after averaging the predicted log probabilities over an ensemble of 10 best models for each noise scale, shown in *SI Appendix*, Fig. S11. For the T4 Lysozyme, the Pearson and the Spearman correlations with the experimental ΔΔG values are reported, when only using the wild-type structure $\Delta \log P_{\mathrm{wt}}$, and when using both the wild-type and the mutant structures of the 40 T4 Lysozyme variants ΔlogP (Fig. 4). For the SARS-CoV-2 task, the AUC for classifying mutations into bound vs. unbound is reported (Fig. 5). Bold values indicate the best performance for each metric.

### H-CNN Predicts Fitness Effect of Mutations for Binding of SARS-CoV2 to the ACE2 Receptor.

Recent deep mutational scanning (DMS) experiments measured the effect of thousands of mutations in the receptor-binding domain (RBD) of SARS-CoV-2 on the folding of the RBD (through expression measurements) and its binding to the human Angiotensin-Converting Enzyme 2 (ACE2) receptor (63, 64).

H-CNN can be used to predict the effect of mutations on RBD, either in isolation or bound to the ACE2 receptor. The former can be interpreted as the effect of mutations on the stability of RBD, which is measured by the expression of the folded domain in the experiments (63–65), while the latter can be used to characterize amino acid preferences for binding at the RBD-ACE2 interface. Fig. 5 *A* and *B* shows that the H-CNN predictions are correlated with the stability and binding measurements in the DMS experiments from ref. 64; site-specific effects are depicted in *SI Appendix*, Figs. S13 and S14.

The average effect of mutations on expression and binding can define three categories of sites and/or mutations (Fig. 5*C*): i) sites that are intolerant to mutations (due to destabilizing effects) and show a substantially reduced expression of mutants (blue), ii) sites that are tolerant of mutations for expression but not binding (green), and iii) sites that are tolerant of mutations for both expression and binding (pink). Using the isolated structure of RBD, H-CNN can well classify mutations according to their stability effect (AUC = 0.80; Fig. 5*D*). Similarly, with the structure of the RBD-ACE2 complex, H-CNN can classify mutations according to their tolerance for binding (AUC = 0.77; Fig. 5*D*).

Expectantly, the sites that are tolerant of mutations for expression but not binding (green category from the DMS data in Fig. 5*C*) are located at the interface of the RBD-ACE2 complex, and H-CNN correctly predicts this composition (Fig. 5*E* and *SI Appendix*, Fig. S12). The overall impact of mutations on binding for these sites is shown in Fig. 5*E*.

Identifying candidate sites that can tolerate mutations and can potentially improve binding is important for designing targeted mutagenesis experiments. Instead of agnostically scanning single point and (a few) double mutations over all sites, these predictions can inform experiments to preferentially scan combinations of viable mutations on a smaller set of candidate sites. In previous work, evolutionary information was used to design such targeted mutagenesis for the HA and NA proteins of influenza (66, 67). A principled structure-based model could substantially improve the design of these experiments.

In contrast to our stability predictions, noising the training data does not improve the performance of H-CNN in predicting the mutational effects in these DMS experiments (Table 2 and *SI Appendix*, Fig. S15). However, we see performance increases when estimating the mutational effects by averaging over ensembles of best-performing networks (*SI Appendix*, Fig. S15). When comparing to other structure-based models (18, 20, 34), all methods appear to perform comparably well, with MutCompute (34) showing a slightly enhanced performance in classifying mutations that impact the binding of RBD to the ACE2 receptor (Table 2). However, a systematic analysis on a larger set of proteins would be necessary to benchmark these methods for their performance in predicting the effect of mutations on protein stability and function.

### Discussion.

The success of AlphaFold has demonstrated the power of machine learning in predicting protein structure from sequence (3). The challenge now is to leverage the experimentally and computationally determined protein structures to better understand and predict protein function. Our H-CNN model is a computationally powerful method to represent protein tertiary structures and characterizes local biophysical interactions in protein microenvironments. Our model is physically motivated in that it respects rotational symmetry of protein structure data, allowing for significantly faster training time compared to previous approaches (33, 34).

Similar to recent language models, H-CNN also demonstrates strong cross-task generalization by predicting quantitative effects of amino acid substitutions on zero-shot predictions of function, including protein stability or binding of protein complexes. Generally, massive language models trained on large and diverse protein sequence databases are shown to generalize well to predict mutational effects in proteins without any supervision (6, 7, 10, 68, 69). State-of-the-art methods include ESM-1b for zero-shot predictions (10) and MSA transformers that use evolutionary information from MSAs of protein families to predict the effect of mutations (68). The benchmark for these methods is the large set of DMS experiments, for which most zero-shot sequence-based predictions show an average accuracy of about 50% in predicting the rank order of the mutational effects (69). Our structure-based H-CNN method shows a comparable accuracy in predicting the mutational effect in DMS experiments of the RBD protein in SARS-CoV-2, yet with much fewer parameters; a more systematic analysis would be necessary to compare these different approaches. Moreover, in classifying mutations into bound vs. unbound, we show that H-CNN performs comparably well to other zero-short structure-based models (18, 20, 34). Nonetheless, it would be interesting to see how the features extracted by H-CNN can complement the sequence-based language models to potentially improve zero-shot predictions for mutational effects in proteins.

Training H-CNN with noised structure data leads to a substantial improvement in our predictions for the stability effect of mutations, despite a reduced accuracy in network performance. Indeed, ProteinMPNN has previously demonstrated the value of adding noise to the structure data to achieve more robust models for proteins (20). It appears that networks trained on crystal structures may rely on the exact shape of the amino acids in a crystal, which is more constrained than in natural conditions. Adding noise to the training data could reduce this bias, resulting in better generalizations for predicting physicochemical properties of proteins in natural conditions.

Recent work has shown that combining structural data with evolutionary information from MSAs in deep learning models can be powerful in predicting mutational effects in proteins (70). We have shown that H-CNN recapitulates the functional information reflected in evolutionary data, further reinforcing the idea that physically guided structure-based machine learning models could be sufficient in predicting protein function, without a need for MSAs. Importantly, our MSA-independent approach enables us to apply H-CNN to protein structures with no available homologs, including the de novo protein structures.

The H-CNN learned representations of amino acid neighborhoods could be used as input to a supervised algorithm to learn a more accurate model for mutational effects in proteins; a similar approach has been used to model the stability effect of mutations in ref. 18. Moreover, the all-atom representation of protein structures used to train H-CNN allows for generalizability, e.g., using the inferred model to analyze non-amino acid molecules or extending the model and accommodate other elements to study protein–drug or protein–DNA interactions.

Solving the inverse protein folding problem by designing a sequence that folds into a desired structure is a key step in protein design. Recent deep learning methods, including ProteinMPNN (20) and transformer-based ESM-IF1 (12, 19), have shown promise in designing viable sequences with a desired fold for de novo proteins. H-CNN’s ability to learn an effective potential in protein microenvironments merits investigation as to whether similar techniques can be used to solve the inverse folding problem for de novo proteins.

The learned representation of protein microenvironments with H-CNN enables us to characterize the preferences of different amino acid compositions in a structural neighborhood. Additionally, these rotationally equivariant representations could be used as building blocks of larger protein structure units, e.g., to characterize how different molecular features on a protein surface could determine its interactions with other proteins. A study in this direction could shed light on the structure-to-function map of the protein universe.

## Materials and Methods

### Data Preparation.

H-CNN is trained on ProteinNet’s 30% sequence identity splitting of PDB structures available at the time of CASP12 (71); *SI Appendix*, Fig. S6*C* shows that H-CNN performance is not strongly sensitive to the exact splitting of the data. Since structure IDs were not available for the testing set, we used ProteinNet’s training set with 80/20% split as our training and validation sets and ProteinNet’s validation set as our testing set. We further restricted our training/validation to only x-ray crystal structures with resolution of 2.5 Å or better. We also removed any structures from both training and validation set that shared the same UniProtKB accession as T4 Lysozyme and SARS-CoV2 RBD in anticipation of testing the model on downstream tasks. Ultimately, this resulted in 10,957 training structures, 2,730 validation structures, and 212 testing structures. All residues in each structure were used in each set resulting in 2,810,503, 682,689, and 4,472 neighborhoods, respectively.

In addition to using ProteinNet’s splits, separate H-CNN models are also trained, validated, and tested on the splits introduced in the training of ProteinMPNN (20). These splits differ from ProteinNet in that they are split based on both sequence similarity and structural similarity as defined by CATH (72). Following ProteinMPNN, we used a 80/10/10 splitting of the ProteinMPNN clusters taking only one representative per cluster for training, validation, and test sets. This resulted in 14,052 training structures, 1,754 validation structures, and 1,756 testing structures. All residues in each structure were used in each set resulting in 3,331,033, 421,578, and 415,360 neighborhoods respectively. Classification accuracy of H-CNN trained on these splits yielded 68% accuracy (*SI Appendix*, Fig. S4), consistent with the results obtained from the ProteinNet’s splits in Fig. 2*A*.

### Holographic Encoding of Amino Acid Neighborhoods.

We define a residue’s atomic neighborhood N as all atoms within a 10 Å of the central residue’s α-carbon excluding the atoms belonging to the central residue. This point cloud defines a density

[1] $$\begin{array}{c} \rho^{c}\left(r\right)=\sum_{i\in N}v_{i}^{c}\delta^{\left(3\right)}\left(r-r_{i}\right), \end{array}$$

where $r_{i}$ is the coordinate vector of the ith neighbor atom with respect to the central residue’s α-carbon and $v_{i}^{c}$ is a feature vector that describes the physical and chemical properties of each atom, with channels: c∈{carbon, nitrogen,oxygen, sulfur, hydrogen, charge, SASA}.

We project this atomic density onto an equivariant basis via a spherical Fourier transform

[2] $$\begin{array}{c} {\hat{Z}}_{n\ell m}^{c}=\int \rho^{c}\left(r\right)Y_{\ell m}\left(\theta ,\phi \right)R_{n\ell }\left(r\right)\;\;\text{d}\Omega , \end{array}$$

where $Y_{\ell m}\left(\theta ,\phi \right)$ is the spherical harmonic of degree ℓ and order m and $R_{n\ell }$(r) is the radial Zernike polynomial which is nonzero for only nonnegative integer values of the frequency (n−ℓ)/2. We term this projection as holographic encoding of the data; see *SI Appendix* for details.

### Network Architecture and Training.

The neural network that processes the Fourier transformed (holographic) inputs is composed of three operations: i) linearity, ii) Clebsch–Gordan nonlinearity, and iii) spherical batch normalization; see *SI Appendix* for complete details on these operations. In brief, the linearity linearly combines information that transforms similarly under rotations, the Clebsch–Gordan nonlinearity decomposes products back into the equivariant basis, and the spherical batch norm normalizes activations by invariant quantities preserving equivariance of all activations. The Clebsch–Gordan coefficients impose constraints on the m values to use in the decomposition of any product of inputs of given spherical orders $\ell_{1}$ and $\ell_{2}$. However, no constraints are imposed which orders to use or which channels $c_{1},c_{2}$ or radial frequencies to use $n_{1},n_{2}$. Two possible choices were studied in the networks presented here. Simply connected networks, which only take products for $\ell_{1}=\ell_{2},c_{1}=c_{2},n_{1}=n_{2}$, and fully connected networks, which take products between all possible combinations of $\ell_{1},\ell_{2},c_{1},c_{2},n_{1},n_{2}$ in any given layer. Hyperparameter optimization was performed separately for the fully connected and simply connected networks (*SI Appendix*, Fig. S3). The network used throughout this paper is a fully connected network due to its superior performance; see *SI Appendix* and *SI Appendix*, Fig. S2 for specific architecture details.

## Supplementary Material

Appendix 01 (PDF)

## Data Availability

All codes and references to data are available on GitHub through: https://github.com/StatPhysBio/protein_holography (73). All other data are included in the manuscript and/or *SI Appendix*.
